# MCM2 in human cancer: functions, mechanisms, and clinical significance

**DOI:** 10.1186/s10020-022-00555-9

**Published:** 2022-10-27

**Authors:** Yaoqi Sun, Zhongping Cheng, Shupeng Liu

**Affiliations:** 1 Department of Obstetrics and Gynecology, Shanghai Tenth People’s Hospital, Tongji University School of Medicine, Shanghai, 200072 China; 2 Department of Obstetrics and Gynecology, Putuo District People’s Hospital of Shanghai City, Shanghai, 200060 China

**Keywords:** MCM2, DNA replication, Tumor, Biomarker, Immunotherapy

## Abstract

**Background:**

Aberrant DNA replication is the main source of genomic instability that leads to tumorigenesis and progression. MCM2, a core subunit of eukaryotic helicase, plays a vital role in DNA replication. The dysfunction of MCM2 results in the occurrence and progression of multiple cancers through impairing DNA replication and cell proliferation.

**Conclusions:**

MCM2 is a vital regulator in DNA replication. The overexpression of MCM2 was detected in multiple types of cancers, and the dysfunction of MCM2 was correlated with the progression and poor prognoses of malignant tumors. According to the altered expression of MCM2 and its correlation with clinicopathological features of cancer patients, MCM2 was thought to be a sensitive biomarker for cancer diagnosis, prognosis, and chemotherapy response. The anti-tumor effect induced by MCM2 inhibition implies the potential of MCM2 to be a novel therapeutic target for cancer treatment. Since DNA replication stress, which may stimulate anti-tumor immunity, frequently occurs in MCM2 deficient cells, it also proposes the possibility that MCM2 targeting improves the effect of tumor immunotherapy.

## Background

Dysregulation of DNA replication has been at the forefront of cancer research, and targeting DNA replication has been a classical chemotherapeutic strategy. Drugs interfering with DNA replication, such as platinum, taxanes, nucleoside and nitrogenous base analogs, topoisomerase inhibitors, and DNA-alkylating agents, account for the majority of clinically used chemotherapeutics and form the cornerstone for a number of new molecular-targeting therapies (Dobbelstein and Moll 2014; Browning et al. 2017). The minichromosome maintenances (MCMs) are the best-known proteins involved in the initiation of DNA replication and are extremely important in maintaining genomic stability (Neves and Kwok 2017; Wang et al. 1874). The six conserved proteins, including MCM2, MCM3, MCM4, MCM5, MCM6, and MCM7, form a hexameric ring-shaped complex, which acts as a DNA helicase that unwinds duplex DNA (Maiorano et al. 2006). Aberrant expression and activation of MCM2-7 directly impair DNA replication, cause genomic instability, and were correlated with tumorigenesis and malignancies (Pruitt et al. 2007; Wu et al. 2018).

The overexpression of MCMs was detected in various cancers, such as breast cancer, hepatocellular carcinoma (HCC), and non-small cell lung cancer (NSCLC), suggesting their role in cancer development and diagnosis (Ha et al. 2004; Issac et al. 2019; Liu et al. 2018; Zhou et al. 2021; Li et al. 2019; Stockley et al. 2020; Toyokawa et al. 2011; Qu et al. 2017; Su 2022; Semple and Duncker 2004). MCM2 is a critical regulator in the DNA replication initiation (Tsuji et al. 2006). Its deficiency was related to tumorigenesis in mice (Pruitt et al. 2007). And the dysfunction of MCM2 was found to be correlated with the progression and poor prognoses of many cancer types, such as lung cancer (Ramnath et al. 2001; Huang et al. 2021), HCC (Tang et al. 2022; Quaglia et al. 2006), cervical cancer (He et al. 2020; Shroyer et al. 2006), breast cancer (Issac et al. 2019; Gonzalez et al. 2003), oral squamous cell carcinoma (Kodani et al. 2003; Scott et al. 2006), prostate cancer (Toubaji et al. 2012; Long et al. 2020) and pancreatic cancer (Deng et al. 2020; Peng et al. 2016). Targeting MCM2 with a small-molecular inhibitor showed a potent anti-tumor efficacy in NSCLC (Lin et al. 2020). These findings indicate that MCM2 may be not only a diagnostic or prognostic marker but also a potential therapeutic target in multiple cancers. In this review, the structure and biological function of MCM2 will first be summarized, followed by its role in cancer development, diagnosis, and prognosis. Its potential to be a therapeutic target will also be discussed.

## MCM2: mechanism and function in DNA replication

### Anatomy of MCM2

The MCM2 gene, previously known as BM28 or CDCL1, is located on 3q21 and includes 17 exons (Mincheva et al. 1994; Nakatsuru et al. 1995). Three alternatively spliced transcript variants have been found, including two linear mRNA and a non-coding RNA. The functional protein of MCM2 is encoded by transcript variant 1 (NM_004526.4) with 3434 base pairs. 16 coding exons in this transcript variant encode a 904 amino acids polypeptide with a predicted molecular mass of 99 kDa. The migration of MCM2 on SDS-gels revealed a 125 kDa polypeptide, which may be attributed to the irregular rate on SDS-gels caused by different amino acid sequence features (Todorov et al. 1994). MCM2 protein has three domains, including the N-terminal domain, central AAA + domain, and C-terminal domain, which is similar to other MCM proteins. The AAA + domain, containing about 250 amino-acid residues, is highly conserved and responsible for the catalytic activity of MCM2 (Costa and Onesti 2009). The zinc finger motif (CX_2_CX_19_CX_2_C) in the N-terminal domain plays a critical role in the interaction between MCM2 and other MCM proteins (Tye 1999; Zhai et al. 2017). In addition, both MCM2 and MCM3 contain the nuclear localization signaling (NLS) sequences, which are necessary for the nuclear translocation of MCM2, MCM3, or other MCMs (Liku et al. 2005; Pasion and Forsburg 1999). A large number of studies have been carried out to explore the structure and function of MCM2. Detailed findings have been recently reviewed by Yeon-Soo Seo and Young-Hoon Kang (Seo and Kang 2018).

### Role of MCM2 in DNA replication initiation

**Fig. 1:**
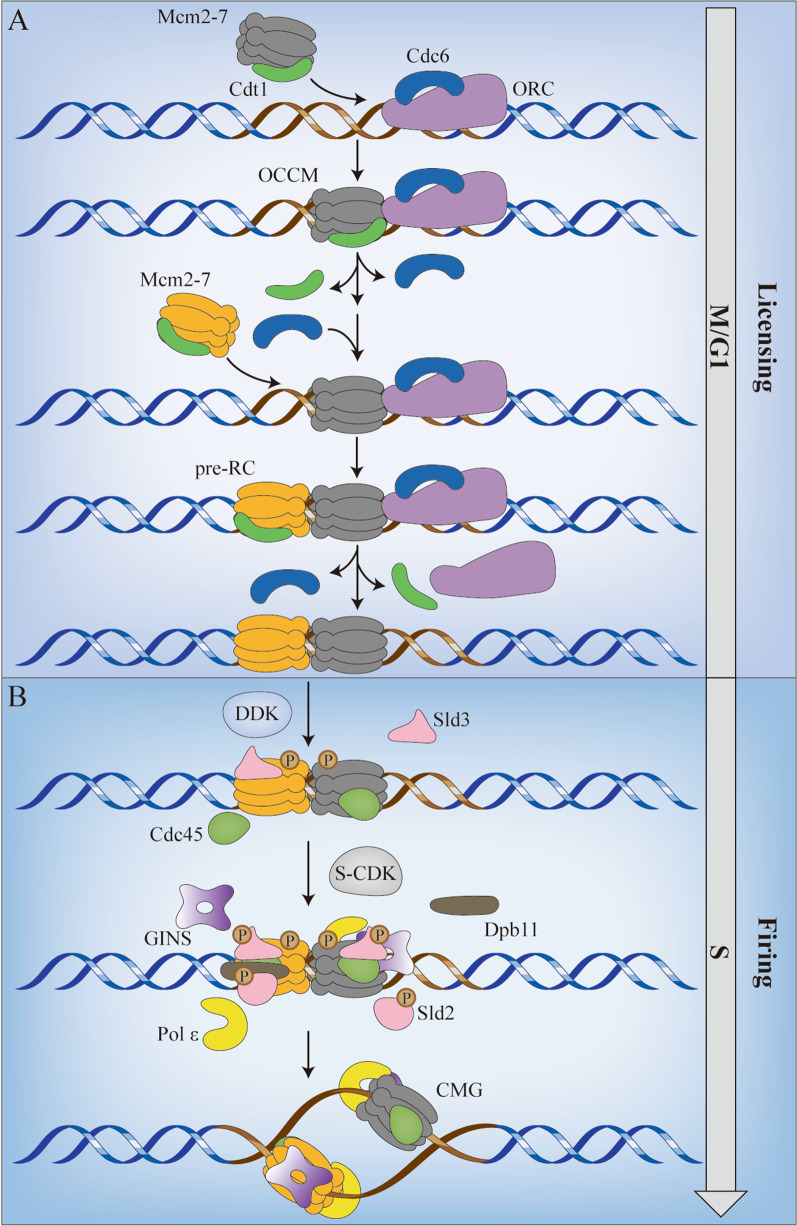
MCM2 in DNA Replication. The two-step mechanism ensures a single round of replication. **A** The loading of Mcm2-7 only occurs in late M/G1 phase. **B** The activation of Mcm2-7 is mediated by DDK and S-CDK in S phase

In eukaryotic cells, MCM2-7, the replicative DNA helicase, plays an important role in ensuring a single round of replication, with no region of the genome left unreplicated or replicated more than once (Blow and Dutta 2005). The highly coordinated process, which is supported by a two-step mechanism, was illustrated in the budding yeast system (Remus et al. 2009) (Fig. 1). In the first step, also known as licensing process, the six-subunit origin recognition complex (ORC) binds to DNA to mark the sites of replication initiation, followed by the recruitment of cell division cycle 6 (Cdc6) (Ticau et al. 2015). Escorted by chromatin licensing and DNA replication factor 1 (Cdt1), the first Mcm2-7 hexamer is recruited by the ORC-Cdc6 complex and encircles the double-stranded DNA in an ATP-dependent reaction, thus forming the ORC-Cdc6-Cdt1-Mcm2-7 (OCCM) complex (Zhai et al. 2017). After the orderly release of Cdc6 and Cdt1, Cdc6 again binds to ORC, and the second Cdt1·Mcm2-7 heptamer is recruited by the first Mcm2-7 hexamer to complete a head-to-head double hexamer, facilitating bidirectional replication initiation. The pre-RC complex is assembled, but the helicase remains inactive (Blow and Dutta 2005; Ticau et al. 2015; Yuan et al. 2017). The second step, known as firing, is triggered by the activation of S-phase cyclin-dependent kinase (S-CDK) and Dbf4 dependent Cdc7 kinase (DDK) (Heller et al. 2011). DDK phosphorylates Mcm2, Mcm4 and Mcm6, and recruits Cdc45 and Sld3 to the double hexamer (Heller et al. 2011; Saleh et al. 2022). Subsequently, Sld2 and Sld3, phosphorylated by S-CDK, bind to Dpb11 and facilitate the loading of GINS and polymerase ε to the replication initiation (Tanaka et al. 2007). Together, these events contribute to the formation of the Cdc45/Mcm2-7/GINS (CMG) complex, which is required for DNA unwinding. During the initiation of DNA replication, S-CDK appears to have multiple effects on preventing re-initiation, including the nuclear exclusion of Cdt1 and Mcm2-7, phosphorylation of ORC, and suppression of Cdc6 (Blow and Dutta 2005; Nguyen et al. 2001). The low level of S-CDK in late mitosis and G1 phase facilitates pre-RC’s assembly, while the high level of S-CDK in S phase promotes the activation of DNA helicase and prevents re-loading of Mcm2-7 (Blow and Dutta 2005).

### Regulation of MCM2 in DNA replication

Given the critical function of MCM2 in DNA replication, the mechanisms that control and modulate the activity of MCM2 are complex in this process. Current studies mainly focus on the effects of MCM2 protein modification on its activity and function. And multiple regulatory regions are distributed on MCM2 protein. It was reported that aberrant phosphorylation of MCM2 could lead to the dysfunction of DNA replication (Tsuji et al. 2006; Bonda et al. 2009; Montagnoli et al. 2006). Toshiya Tsuji et al. found three phosphorylation sites of MCM2 (Ser27, Ser41, and Ser139) by Cdc7/Dbf4 both in vitro and in vivo, which plays a critical role in the initiation of DNA replication but has no effects on chromatin loading of MCM2 (Tsuji et al. 2006). Alessia Montagnoli et al. also identified several phosphorylation sites in the MCM2 N-terminal region, including three sites (Ser40, Ser 53, and Ser108) by Cdc7, three sites (Ser13, Ser27, and Ser41) by CDK, and one site (Ser139) by casein kinase 2 (CK2). In addition, hydroxyurea (HU) could induce hyperphosphorylation of MCM2 at Ser40/53/108 and consequently prevent the disassociation of MCM2 from the chromatin (Montagnoli et al. 2006). Ser108 was also reported to be one of the phosphorylation sites of Ataxia telangiectasia and Rad3-related (ATR) (Cortez et al. 2004; Martinez et al. 2014). The overlapping regulatory site of Cdc7 and ATR seems to ensure the accuracy and integrity of DNA replication under replication stress. Although many phosphorylation sites of MCM2 have been identified in vitro, the biological functions of various sites remain unascertained (Fei and Xu 2018).

## MCM2 in cancer development

There are an excess number of dormant origins licensed by loading MCM2-7 on chromatin, playing a vital role in maintaining genomic stability, especially serving as a backup system to protect cells from replication stress (Ibarra et al. 2008). Insufficient MCM2-7 caused genomic instability and impaired cell cycle progression, leading to early-onset cancer (Chuang et al. 2010).

### MCM2 deficiency induces tumorigenesis

**Fig. 2:**
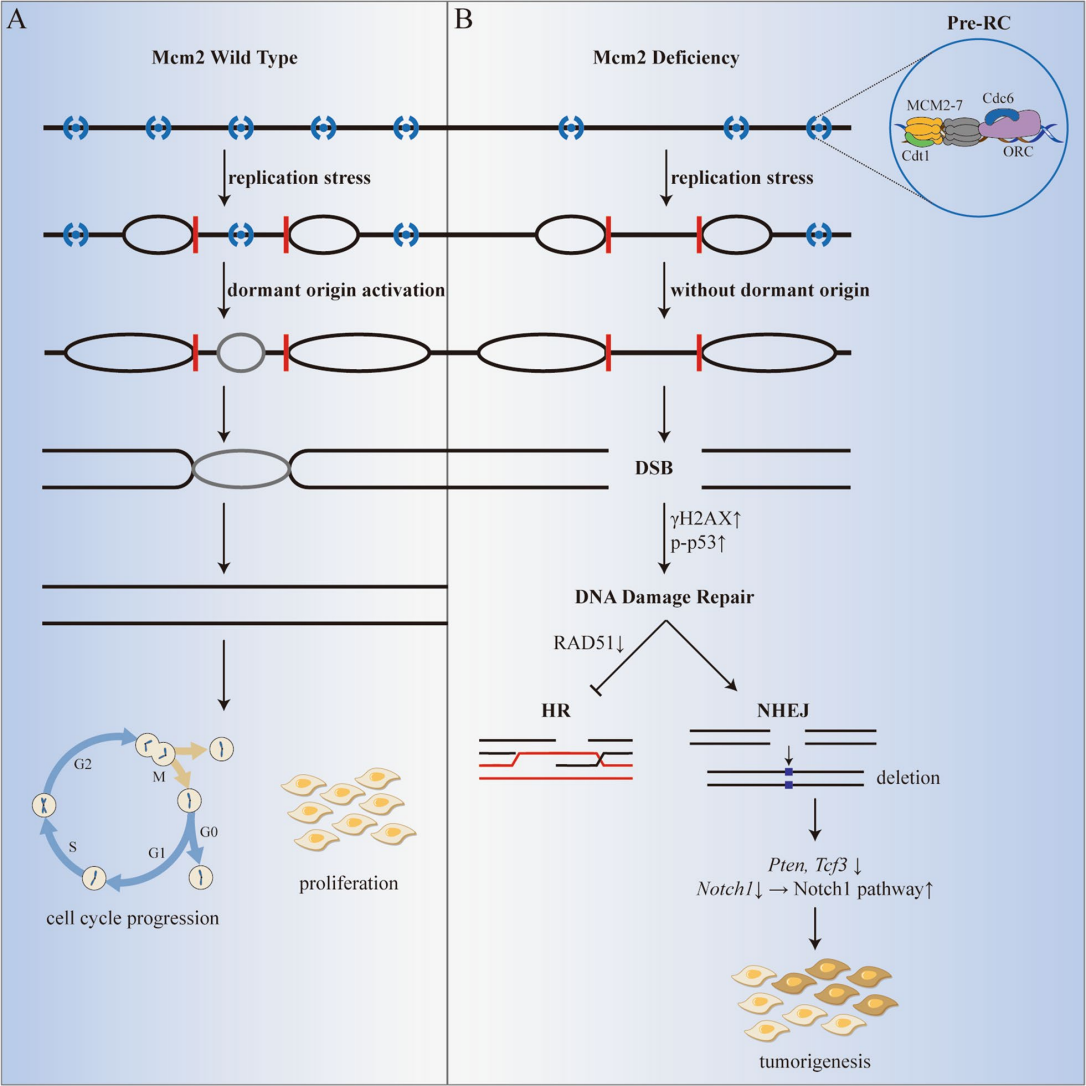
MCM2 Deficiency and Tumorigenesis. Under replication stress, (**A**) the excess number of dormant origins maintains genomic stability and promotes cell cycle progression in Mcm2 wild-type mice, (**B**) the deficiency of Mcm2 leads to the reduction of dormant origins, the increase of DSBs, and the onset of cancer

The correlation between Mcm2 deficiency and tumorigenesis was first reported 15 years ago by observation of a higher incidence of lymphoma in Mcm2^IRES−creERT2/IRES−creERT2^ (Mcm2^cre/cre^) mice (Pruitt et al. 2007). The introduction of IRES-creERT2 caused the reduction of Mcm2 level to approximately one-third of wild type, leading to the decrease of dormant origins (Pruitt et al. 2007; Kunnev et al. 2010). Dimiter Kunnev et al. demonstrated that the normal growth and DNA replication were not influenced significantly in Mcm2 deficient cells unless under replication stress (Kunnev et al. 2010). Consistent with this, Mcm2^cre/cre^ mice showed no symptoms when they are young, but succumbed to T- or B- cell lymphoma within four months of age (Pruitt et al. 2007). The level of phosphorylated-H2A histone family member X (γH2AX) and phosphorylated p53 increased in cells derived from Mcm2^cre/cre^ mice compared with those from Mcm2^wt^ mice (Pruitt et al. 2007; Kawabata et al. 2011). These findings confirmed the accumulation of double-strand DNA breaks (DSBs) in Mcm2 deficient cells, which may be attributed to the impairment of dormant origins recruiting and firing under replicative stress. Jun Huang et al. reported that RAD51, a critical homologous recombination (HR) protein, directly interacted with MCM2 in HCT116 cells. The downregulation of MCM2 and MCM6 reduced RAD51 chromatin fraction and foci forming, or rather impeding the HR mediated by RAD51 (Huang et al. 2018; Scully et al. 2019). Michael E. Rusiniak et al. reported the high frequency of *Pten* and *Tcf3* deletions, and activation of Notch signaling pathway in Mcm2 deficient mice (Rusiniak et al. 2012). Such gene-rich, early replicating regions of genome were demonstrated to be more sensitive to Mcm2 deficiency (Kunnev et al. 2015). The phenotype was further confirmed by Mianmian Yin et al., who found that DSBs repaired by non-homologous end joining (NHEJ) in Mcm2^cre/cre^ mice cause indel mutations and structural variations. Copy number alteration (CNA) analysis confirmed the homozygous deletions of *Pten* and *Tcf3*, and partial deletions of *Notch1,* resulting in *Notch1* activation (Yin et al. 2019). It seems that HR repair of DSBs is impaired in Mcm2 deficient cells and NHEJ causes the mutations of both oncogenes and tumor suppressor genes. These mutations, including deletions of tumor suppressor genes and activation of oncogenic pathways, lead to the susceptibility of Mcm2^cre/cre^ mice to cancer (Fig. 2).

### MCM2 promotes cancer progression

**Fig. 3:**
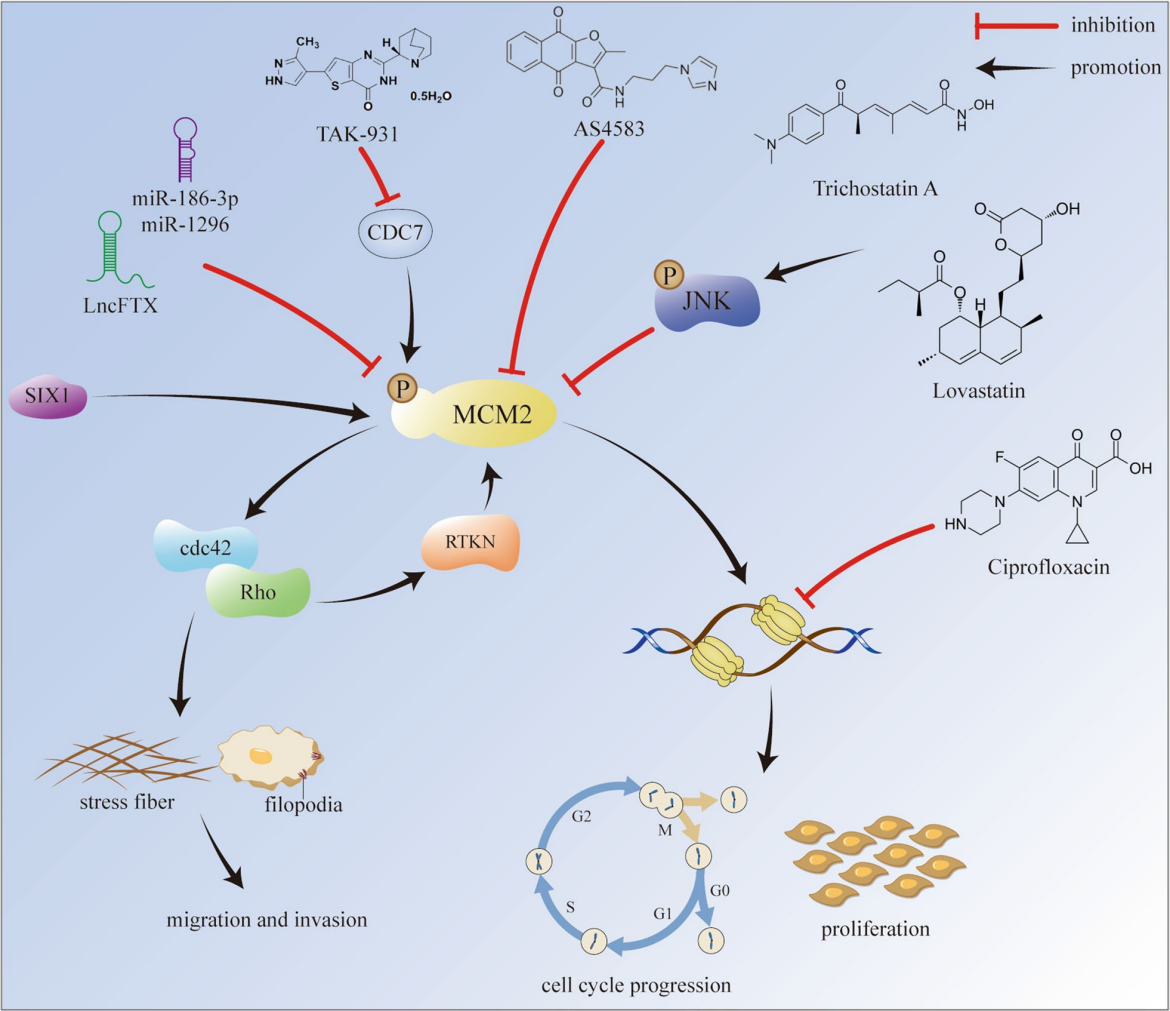
Potential mechanisms of MCM2 in promoting cancer progression and existing inhibitors targeting MCM2

Proliferation, migration, invasion, and metastasis are the characteristics of cancer progression. MCM2 is a proliferative marker and positively correlates with TNM stage and lymph node metastasis in many cancers (Wu et al. 2018; Toubaji et al. 2012; Wu and Xi 2021). Xiurong Lu et al. found that MCM2 promoted cell proliferation and inhibit cell apoptosis in cervical cancer (Lu et al. 2021). Cuiling Wang et al. reported that MCM2 overexpression facilitated cholangiocarcinoma (CCA) progression via suppressing p53 signaling pathway. Increased level of MCM2 accelerated proliferation, migration, and invasion of tumor cells, and inhibited apoptosis (Wang et al. 2022). K-M Lau et al. reported that upregulation of MCM2 promoted anchorage-dependent and -independent growth, migration, and invasion in medulloblastoma (MB) cells. The knockdown of MCM2 suppressed the activation of cdc42 and Rho, which impaired the formation of filopodia and stress fibers and thereby reduced migration and invasion of MB cells (Lau et al. 2010). Interestingly, GuoQiang Qu et al. observed that rhotekin (RTKN), a Rho effector protein, could upregulate the expression of MCM2 to increase cell proliferation, migration, and invasion in colon cancer (Qu et al. 2015). It suggests that there may be feedback regulation between MCM2 and Rho. Gulinisha Aihemaiti et al. reported that the role of MCM2 in cancer progression was dependent on the subcellular location of MCM2. High expression of cytoplasmic MCM2 exhibited a higher level of DNA damage-induced apoptosis in ovarian cancer cells and demonstrated excellent prognosis in patients with ovarian clear cell carcinoma (Aihemaiti et al. 2018). F Liu et al. found that lnc-FTX, an X-inactive-specific transcript regulator, impeded DNA replication and inhibited cell growth in HCC. According to RNA immunoprecipitation, lnc-FTX bonded to MCM2 and prevented MCM2 from loading onto chromatin, thus inhibiting proliferation (Liu et al. 2016). Dan Liu et al. revealed that sine oculis homeobox homolog 1 (SIX1) enhanced the MCM2 level to further accelerate the initiation of DNA replication and cell cycle in cervical cancer (Liu et al. 2014). In summary, MCM2 has the potential to promote cancer progression. Nevertheless, more efforts are needed to explore the downstream mechanisms of MCM2 in facilitating proliferation, migration, and invasion (Fig. 3).

### MCM2 and cancer stem cells

Cancer stem cells (CSCs) are thought to be the seed of tumor development, relapse, and chemoresistance (Lupia and Cavallaro 2017). Steven C. Pruitt et al. found that stem/progenitor cells were deficient in various tissues in Mcm2^cre/cre^ mice. Based on the staining of IdU and CIdU on histological sections, the number of Mcm2^+^ stem cells was reduced in Mcm2^cre/cre^ mice compared with wild-type mice, but not the rate of division of progenitor cells (Pruitt et al. 2007). It suggests the critical role of Mcm2 in the maintenance of stem/progenitor cell viability and function (Pruitt et al. 2007). Yitong Zhang et al. found that MCM2 was one of the key genes correlated to the characteristics of lung adenocarcinoma (LUAD) stem cells via WGCNA according to the stemness index based on mRNA expression (mRNAsi) index (Zhang et al. 2020). MCM2 was overexpressed in CSCs from glioblastoma (GBM), colon cancer, and breast cancer (Abe et al. 2015; Deleyrolle et al. 2011; Wang et al. 2020). Shinya Abe et al. found frequent co-localization of MCM2 and CSCs markers, including CD133 and ALDH-1, in triple-negative breast cancer (TNBC) (Abe et al. 2015). Longgang Wang et al. observed that the activation of NF-κB downregulated the expression of miR-195-5p/497-5p, which suppressed the tumorigenesis and impaired the stemness of colon cancer stem cells (CCSCs) by targeting MCM2 (Wang et al. 2020). Taken together, these results indicate that MCM2 has tremendous potential in maintaining the viability and promoting stem-like properties of CSCs.

## MCM2 as a cancer biomarker

MCM2 is a sensitive biomarker for cancer cell proliferation in keeping with classical markers proliferating cell nuclear antigen (PCNA) and Ki-67, and performs even better in colorectal cancer (Hanna-Morris et al. 2009), breast cancer (Yousef et al. 2017; Joshi et al. 2015), and esophageal squamous cell carcinoma (ESCC) (Kato et al. 2003). Many cancers showed a positive correlation between MCM2 expression levels and malignant progression of cancers in accordance with the inherent function of MCM2 in cell proliferation (Wu et al. 2018; Wu and Xi 2021; Kato et al. 2003; Mehdi et al. 2016; Liu et al. 2017; Cobanoglu et al. 2010; Giaginis et al. 2009). It is rational that MCM2 may serve as a reliable diagnostic and prognostic marker in cancers.

### MCM2 as diagnostic and prognostic markers

MCM2 was overexpressed in a number of cancers and was extensively used as a broad-spectrum diagnostic and prognostic biomarker (Table 1).

**Table 1:** MCM2 as a cancer biomarker

Cancer Type	Expression in cancer	Diagnostic Marker	Prognostic Marker	References
Breast cancer	Overexpress	√	√	Issac et al. (2019); Gonzalez et al. (2003); Wojnar et al. (2011); Cheng et al. (2020); Liu et al. (2021); Tőkés et al. (2016)
Cervical cancer	Overexpress	√	√	He et al. (2020); Shroyer et al. (2006); Tang et al. (2018); Liao et al. (2018); Kelly et al. (2006); Siddiqui et al. (2008); Boucher et al. (2009); Ding et al. (2020); Aximu et al. (2009; Sanati et al. (2010)
Cholangiocarcinoma	Overexpress	–	√	Wang et al. (2022)
Colorectal cancer	Overexpress	√	–	Davies et al. (2002; Wang et al. (2009)
Cutaneous squamous cell carcinoma	Overexpress	√	–	Rymsza et al. (2022)
Endometrial carcinoma	Overexpress	–	√	Lan et al. (2021)
Esophageal adenocarcinoma	Overexpress	√	–	Sirieix et al. (2003)
Gastric carcinoma	Overexpress	–	√	Tokuyasu et al. (2008)
Glioma	Overexpress	–	√	Gao et al. (2019); Hua et al. (2014)
Hepatocellular carcinoma	Overexpress	√	√	Tang et al. (2022); Quaglia et al. (2006); Sang et al. (2018); Sun et al. (2010a); Yang et al. (2018); Marshall et al. (2010)
Laryngeal squamous cell carcinoma	Overexpress	√	–	Chatrath et al. 2003)
Lung cancer	Overexpress	√	√	Ramnath et al. (2001); Huang et al. (2021); Yang et al. (2006); Hashimoto et al. (2004); Sakai et al. (2022); Pan et al. (2020); Zhang et al. (2021); Tan et al. (2001)
Melanoma	Overexpress	√	–	Yan et al. (2016); Boyd et al. (2008)
Oral squamous cell carcinoma	Overexpress	√	√	Kodani et al. (2003); Scott et al. (2006)
Ovarian cancer	Overexpress	–	√	Kulkarni et al. (2007); Gakiopoulou et al. (2007)
Pancreatic cancer	Overexpress	√	√	Deng et al. (2020); Peng et al. (2016)
Prostate cancer	Overexpress	√	√	Toubaji et al. (2012); Long et al. (2020)
Renal cell carcinoma	Overexpress	–	√	Dudderidge et al. (2005); Zhong et al. (2017)
Tongue squamous cell carcinoma	Overexpress	√	–	Li et al. (2008)
Urothelial carcinoma	Overexpress	√	–	Moatamed et al. (2013); Saeb-Parsy et al. (2012)

#### MCM2 in lung cancer

MCM2 was first reported as an independent prognostic marker in NSCLC. Nithya Ramnath et al. examined the expression of MCM2 and Ki-67 via immunohistochemistry in an NSCLC cohort (n = 221). Patients with higher MCM2 levels had a shorter median survival time and a higher relative risk (RR) of death, while Ki-67 showed no significant association between survival and expression level in this cohort (Ramnath et al. 2001). Nevertheless, Jun Yang et al. reported that higher expression of MCM2 showed a non-significant correlation with increased RR of death (p = 0.22) in their NSCLC cohort (n = 128). A combined analysis of MCM2 and gelsolin demonstrated a better predicting performance of survival (Yang et al. 2006). Although subsequent studies confirmed that high level of MCM2 indicated a poor prognosis in both LUAD (Hashimoto et al. 2004; Sakai et al. 2022) and lung squamous cell carcinoma (LUSC) (Pan et al. 2020), a larger cohort is needed to confirm its prognostic value. Bioinformatic analysis revealed that the expression of MCM2 was upregulated in LUAD and LUSC, and associated with the tumor stage (Zhang et al. 2021). Dong-Feng Tan et al. reported that compared with anti-Ki-67, anti-MCM2 detected more proliferative premalignant lung cells near the epithelial surface, which are prone to fall into sputum (Tan et al. 2001). The combination of MCM2, MCM5, and CDC6 showed a sensitivity of 94.4% in diagnosing malignant lung cells (Huang et al. 2021). These studies suggest the potential of MCM2 to be a diagnostic marker of lung cancer.

#### MCM2 in hepatocellular carcinoma

MCM2 was identified as a hypomethylated, highly expressed gene in HCC via bioinformatic analysis (Sang et al. 2018). The lower methylation level of cg08889930, an enhancer of *MCM2*, and higher expression of MCM2 mRNA predicted shorter overall survival (OS) for HCC patients in The Cancer Genome Atlas (TCGA) cohort (Tang et al. 2022). Overexpression of MCM2 was validated to be associated with poor differentiation and malignant progression in HCC via mRNA-seq and tissue microarray analysis (Sun et al. 2010a; Yang et al. 2018). A retrospective study on HCC patients who underwent liver transplantation found that the MCM2 labeling index (LI), highly sensitive measurement of proliferation, was significantly associated with vascular invasion and HCC recurrence (Marshall et al. 2010). Alberto Quaglia et al. found that the co-expression of Ki-67, MCM2, and geminin could help evaluate the progression from cirrhosis to HCC by examining cirrhosis and HCC samples from 5 patients (Quaglia et al. 2006). Jing Yang et al. designed an MCM2-targeted NIR-II probe CH1055-MCM2 with excellent imaging properties, which helps the diagnosis of HCC (Yang et al. 2018).

#### MCM2 in breast cancer

The expression of MCM2 correlated positively with the grade of malignancy and negatively with estrogen receptor (ER) and progesterone receptor (PR) expression in invasive ductal breast carcinoma (IDC), which helps distinguish luminal A from luminal B, Erb-B2 receptor tyrosine kinase 2 (HER2)-positive, and TNBC (Issac et al. 2019; Wojnar et al. 2011). The level of MCM2 expression was tightly associated with patient survival in breast cancer. According to a breast cancer cohort (n = 221), patients with a higher level of MCM2 LI experienced early relapse and shortened OS (Gonzalez et al. 2003). Bioinformatic analysis using TCGA and oncomine cohort further confirmed the potential of MCM2 to be a prognostic biomarker in breast cancer (Cheng et al. 2020; Liu et al. 2021).

#### MCM2 in cervical cancer

MCM2 was upregulated in cervical cancer (CC). It appears that high levels of MCM2 were closely associated with longer OS in CC, which may be attributed to different intracellular locations of MCM2 (Aihemaiti et al. 2018; Tang et al. 2018). Most of the studies were based on bioinformatic analysis, IHC and molecular biology experiments are needed to determine the location of MCM2 and interpret the reason why MCM2 plays a protective role in the progression of CC.

Sérgio Menezes Amaro Filho et al. found a strong correlation between the MCM2 expression and human papillomavirus (HPV) infection through IHC and in situ hybridization, respectively (Sawaya et al. 2019). Yu-Cong Li and Guang-Dong Liao et al. found that p16/Ki-67 or p16/MCM2 dual staining performed better than cytology in triaging patients infected with high-risk HPV (hr-HPV) (Liao et al. 2018; Li et al. 2020). Thus, MCM2 was introduced to improve the efficiency of cervical cancer screening. The ProEx C, a cocktail of antibodies that target topoisomerase IIA (TOP2A) and MCM2, was first introduced into cervical cytology by Kenneth R. Shroyer (Shroyer et al. 2006). Compared with routine liquid-based cytology, ProEx C showed higher sensitivity and positive predictive value for high-grade squamous intraepithelial lesions (HSIL) (Kelly et al. 2006). Momin T Siddiqui et al. reported that ProEx C was more sensitive and specific than the hr-HPV DNA test in detecting high-grade cervical intraepithelial neoplasia (CIN) from atypical squamous cell (ASC-US) cytology (Siddiqui et al. 2008). A cocktail of p16^INK4a^ and ProEx C provided the highest diagnostic value for detecting both HSIL and low-grade squamous intraepithelial lesions (LSIL) (Boucher et al. 2009). ProEx C was also reported to be an independent risk factor for LSIL progression into HSIL (Ding et al. 2020). Based on histologic sections, PrcEx C could help distinguish dysplastic squamous and endocervical lesions from neoplastic lesions (Aximu et al. 2009; Sanati et al. 2010). Therefore, ProEx C may be a useful test method to improve the sensitivity and specificity of cervical screening.

### MCM2 as a predictive marker of chemotherapy response

MCM2 was also used for predicting chemotherapy response in several malignancies. Bioinformatic analysis showed the upregulation of MCM2 in T-cell acute lymphoblastic leukemia samples and a negative correlation with the response of 39 drugs (Xia et al. 2019). Tímea Tőkés et al. reported that MCM2, Ki67, cyclin A, and phosphohistone-H3 (PHH3) predicted response to primary systemic therapy in advanced breast cancer patients (Tőkés et al. 2016). The overexpression of these four proliferative markers suggested higher pathological complete remission (pCR) rate but worse prognosis in breast cancer (Tőkés et al. 2020). It was also reported that the MCM2 index was positively correlated with neoadjuvant therapy response in sarcoma (Matsubara et al. 2008). Chanchan Gao et al. suggested that lower expression of MCM2 was associated with a better response to treatment of anti-programmed cell death 1 (PD-1) and cisplatin in small cell lung cancer (SCLC) (Gao et al. 2021). The inconsistent correlation may be ascribed to different regimens and timing of chemotherapy (Korde et al. 2021). Moreover, MCM2 is one of the proliferative markers as well as a co-expression gene with CSCs markers (Abe et al. 2015). And CSCs act as the driving force behind chemoresistance and recurrence (Lupia and Cavallaro 2017). Both proliferative tumor cells and quiescent CSCs should be taken into account when predicting chemotherapy response with the expression of MCM2.

## MCM2 as a cancer therapeutic target

DNA replication is the classic target of many anti-tumor agents. Since MCM2 plays a vital role in DNA replication and is correlated with the progression of many malignancies, it may be a potential target for chemotherapy.

### Knockdown of MCM2 shows anti-tumor effects

Inhibiting MCM2 through miRNA and siRNA suppressed the proliferation of tumor cells in vitro in cancers including cervical cancer (Xue et al. 2021), MB (Lau et al. 2010), GBM (Hu et al. 2022), HCC (Qin and Tang 2004; Sun et al. 2010b), colon cancer (Liu et al. 2013) and lung cancer (Wu et al. 2018; Lin et al. 2020; Cheung et al. 2017; Zhang et al. 2015). MiRNA could inhibit MCM2 through binding to the 3’-UTR of MCM2 mRNA. It was reported that miR-186-3p could inhibit cell proliferation and induce cell apoptosis, and miR-1296 could block S-phase entry by targeting MCM2 (Lu et al. 2021; Majid et al. 2010). Chantal Hoi Yin Cheung et al. detected that silencing of MCM2 could impede cancer cell proliferation via downregulating the phosphorylation of high mobility group AT-hook 1 ^S99^ (HMGA1^S99^) (Cheung et al. 2017). RNAi-mediated depletion of MCM2 induced G2/M-phase arrest in GBM and HCC cells (Hu et al. 2022; Qin and Tang 2004; Sun et al. 2010b), and G1-phase arrest in colon cancer and lung cancer cells (Wu et al. 2018; Liu et al. 2013; Cheung et al. 2017; Zhang et al. 2015). Different cell cycle arrest may be attributed to the heterogeneity of cancer cells and the dysfunction of distinct cell cycle checkpoints (Kyei Barffour and Acheampong 2021; McIntosh and Blow 2012; Saito et al. 2022; Huang et al. 2015). Association of MCM2 knockdown with increased sensitivity to chemotherapy was detected in many cancers. It was reported that RNAi-mediated depletion of MCM2, MCM4, MCM6, and MCM7 enhanced the sensitivity of SCLC cells to cisplatin (Misono et al. 2021). Kenneth Macleod et al. found an increased level of MCM2 in cisplatin-resistant ovarian cancer cell line PE01^CDDP(^Macleod et al. 2005). Minjie Deng et al. subsequently demonstrated that downregulation of MCM2 can promote the carboplatin sensitivity of A2780 cells through upregulation of p53 (Deng et al. 2019). Thus, it suggests that MCM2 inhibitors may also act as a potential candidate for combination chemotherapy. In addition, depletion of MCM2 inhibited migration and invasion in MB and GBM and induced apoptosis in colon cancer and HCC (Lau et al. 2010; Hu et al. 2022; Qin and Tang 2004; Liu et al. 2013; Zhang et al. 2015). However, the underlying mechanisms remain unclear.

### Available MCM2 Inhibitors

Inhibition of MCM2 could also be achieved by several existing drugs (Table 2, Fig. 3). Thiabendazole (TBZ), an anti-microtubule drug, suppressed cell proliferation by downregulating the expression of MCM2 in GBM (Hu et al. 2022). Ciprofloxacin, a fluoroquinolone antibiotic, inhibited the activity of MCM2-7, resulted in delayed cell proliferation and invasion in vitro, and slowed tumor growth in vivo. Moreover, cells with a higher level of MCM2 were more sensitive to ciprofloxacin (Hsu et al. 2021; Simon et al. 2013). Shahana Majid et al. found that trichostatin A (TSA), the first discovered histone deacetylase (HDAC) inhibitor, and genistein, a nontoxic dietary isoflavone, blocked S-phase entry by inhibiting the expression of MCMs, CDT1, CDC7, and CDK2 in prostate cancer cells (Majid et al. 2010). The inhibitory effect of TSA on MCM2 was further confirmed by an RT-PCR array in the colon cancer cell, HCT116. TSA activated the phosphorylated-mitogen-activated protein kinase 8 (p-JNK), while the addition of the JNK inhibitor, SP600125, restored the expression of MCM2. It suggests the involvement of the JNK signaling pathway in the downregulation of MCM2 by TSA treatment (Liu et al. 2013). A similar mechanism was also detected in lovastatin-treated NSCLC cells. JNK pathway activation was involved in the decrease of MCM2 by lovastatin treatment, and the reduction of MCM2 could be restored by a combination of SP600125 and lovastatin (Zhang et al. 2015). Besides, BI-2536 can inhibit MCM2 and MCM10 to suppress cell proliferation in neuroblastoma (Hsieh et al. 2021), metformin can deplete MCM2 and PCNA in 5-fluorouracil resistant colorectal cancer cells (Kim et al. 2017), norcantharidin can induce the degradation of MCM2 and CDC6 in HepG2 cells (Chen et al. 2013), ellagic acid can downregulate the expression of MCM2-7 in HepG2 (Qiu et al. 2021), widdrol can inhibit MCMs proteins in colon adenocarcinoma HT29 cells (Kwon et al. 2010), and TAK-931 can suppress the phosphorylation of MCM2 through targeting CDC7 (Iwai et al. 2019, 2021).

**Table 2:** The available MCM2 inhibitors

Drug	Classification	Targeting mechanism	References
AS4583	Furanonaphthoquinone-based small molecule	Degrade MCM2 in a ubiquitination-dependent manner	Lin et al. (2020)
BI-2536	Polo-like kinase 1 (PLK-1) inhibitor	Downregulate the expression of MCM2 and MCM10	Hsieh et al. (2021)
Ciprofloxacin	Fluoroquinolone antibiotic	Inhibit the activity of MCM2-7	Hsu et al. (2021); Simon et al. (2013)
Ellagic acid	Natural polyphenolic compound	Downregulate the expression of MCM2-7	Qiu et al. (2021)
Genistein	Nontoxic dietary isoflavone	Downregulate the expression of MCMs, CDT1, CDC7, and CDK2	Majid et al. (2010)
Lovastatin	3-hydroxy-3-methylglutatyl CoA (HMG-CoA) reductase inhibitor	Downregulate the expression of MCM2 (by activating the JNK pathway)	Zhang et al. (2015)
Metformin	Biguanide	Downregulate the expression of MCM2 and PCNA	Kim et al. (2017)
Norcantharidin	Cantharidin derivative	Degrade MCM2 and CDC6	Chen et al. (2013)
RJ-LC-07-48	Analog of AS4583	–	Lin et al. (2020)
TAK-931	CDC7 inhibitor	Suppress the phosphorylation of MCM2 (by targeting CDC7)	Iwai et al. (2021, 2019)
Thiabendazole	Anti-microtubule drug	Downregulate the expression of MCM2	Hu et al. (2022)
Trichostatin A	Histone deacetylase inhibitor	Downregulate the expression of MCMs (by activating the p-JNK)	Liu et al. (2013)
Widdrol	Aromatic compound	Downregulate the expression of MCMs	Kwon et al. (2010)

Other MCM2 targeting ways with higher specificity have also been reported. Shinya Abe et al. found that the Friend leukemia virus (FLV) envelope protein, gp70, can interfere with the nuclear translocation of MCM2 through binding to the NLS1 of MCM2, playing a similar role to the specific inhibitor of MCM2. The cytoplasmic gp70-MCM2 complex bound to protein phosphatase 2A (PP2A) and relieved the inhibition of PP2A on DNA-dependent protein kinase (DNA-PK), leading to enhanced DNA-damage-induced apoptosis via the activation of p53/cleaved caspase 3 pathway (Abe et al. 2012). To further improve the MCM2-targeted therapy model, the protein transduction domain (PTD) of Hph-1 was conjugated to gp70 to introduce gp70 into the cytoplasm of breast cancer cells. Combined with doxorubicin, Hph-1-gp70 enhanced cell apoptosis and showed anti-tumor efficacy in cells with high MCM2 expression. CD133-high cells, originally resistant to doxorubicin, expressed a high level of MCM2 and showed sensitivity to doxorubicin in the presence of Hph-1-gp70. Immunohistochemistry confirmed that cancer stem cell markers, such as ALDH-1 and CD133, were frequently expressed and colocalized with MCM2. These suggest that Hph-1-gp70 targeting MCM2 may be effective in TNBC cells and CSC-like breast cancer cells (Abe et al. 2015).

Chia-Yi Lin et al. identified a furanonaphthoquinone-based small molecule, AS4583, as an inhibitor of NSCLC cell proliferation. AS4583 caused the degradation of MCM2 in a ubiquitination-dependent manner, which can be reversed by the proteasome inhibitor, MG132, and cullin inhibitor, MLN4924 (Lin et al. 2020). The protein–ligand docking and further co-immunoprecipitation confirmed that AS4583 bound the N-terminal portion of MCM2, where Gln341 contributed substantially to the hydrogen-bond formation. AS4583 inhibited the formation of the replication fork, induced G1/S arrest in NSCLC cells, and showed potent anti-tumor efficacy in H1975 xenograft tumors. RJ-LC-07-48, a new analog of AS4583, showed more potent inhibitory effects on NSCLC cell viability (Lin et al. 2020). This holds promise in designing anti-tumor therapeutics that specifically target MCM2.

## Conclusion and future directions

Evidence from cells, animals, and clinical studies has clearly demonstrated that MCM2 is involved in both physiological and oncogenic processes. The deficiency of MCM2 was proved to be a driver of tumorigenesis by reducing licensing origins and causing genomic instability, both of which increase cancer susceptibility. However, the overexpression of MCM2 was found in a number of cancer types, positively related to the progression of cancer, and even served as a sensitive biomarker for diagnosis, prognosis, and response prediction. It seems contradictory but may be ascribed to the precise control of the MCM2 level, which is important for maintaining the completion and accuracy of DNA replication and the integrity of the genome. Aberrant expression of MCM2, whether upregulation or downregulation, could both increase cancer risk and promote cancer progression. In addition, there are several issues need to be addressed. Firstly, it is necessary to explore the regulatory mechanisms of MCM2 in malignancies for the development of targeted therapy. Secondly, there are no effective and specific small-molecule inhibitors that directly target MCM2 for clinical application nowadays. Although AS4583 was found to induce the degradation of MCM2 by ubiquitination, the anti-tumor mechanism, safety, and efficacy have not been reported. Thirdly, DNA replication stress could activate tumor-cell-intrinsic immunity (Ubhi and Brown 2019; Coquel et al. 2018; Zhang et al. 2021). Since DNA replication stress frequently occurs in MCM2 deficient cells, it is meaningful to explore whether targeting MCM2 can inhibit proliferation and stimulate anti-tumor immunity simultaneously. It is possible that targeting MCM2 makes cold tumors to be hot and enhances sensitivity of tumor immunotherapy. Most importantly, MCM2 is an indispensable protein in both normal and malignant cells. How to protect normal cells and prevent side effects in the condition of MCM2 inhibition is a difficult but urgent problem in targeted therapy. With the in-depth study of MCM2, we will become more confident to illuminate the role of MCM2 as an effective biomarker and therapeutic target in cancers and even bring translational benefits to patients.

## Data Availability

Not applicable.

## Abbreviations

ASC-US: Atypical squamous cell
ATR: Ataxia telangiectasia and Rad3-related
CC: Cervical cancer
CCA: Cholangiocarcinoma
CCSCs: Colon cancer stem cells
Cdc6: Cell division cycle 6
Cdt1: DNA replication factor 1
CK2: Casein kinase 2
CIN: Cervical intraepithelial neoplasia
CMG: Cdc45/MCM2-7/GINS
CNA: Copy number alteration
CSCs: Cancer stem cells
DDK: Dbf4 dependent Cdc7 kinase
DNA-PK: DNA-dependent protein kinase
DSBs: Double-strand DNA breaks
ESCC: Esophageal squamous cell carcinoma
γH2AX: Phosphorylated-H2A histone family member X
GBM: Glioblastoma
ER: Estrogen receptor
FLV: Friend leukemia virus
HCC: Hepatocellular carcinoma
HDAC: Histone deacetylase
HER2: Erb-B2 receptor tyrosine kinase 2
HMGA1: High mobility group AT-hook 1
HMG-CoA: 3-Hydroxy-3-methylglutatyl CoA
HPV: Human papillomavirus
hr-HPV: High-risk HPV
HR: Homologous recombination
HSIL: High-grade squamous intraepithelial lesions
HU: Hydroxyurea
IDC: Invasive ductal breast carcinoma
LI: Labeling index
LSIL: Low-grade squamous intraepithelial lesions
LUAD: Lung adenocarcinoma
LUSC: Lung squamous cell carcinoma
MB: Medulloblastoma
MCMs: Minichromosome maintenances
NHEJ: Non-homologous end joining
NLS: Nuclear localization signaling
mRNAsi: Stemness index based on mRNA expression
NSCLC: Non-small cell lung cancer
OCCM: ORC-Cdc6-Cdt1-MCM2-7
ORC: Origin recognition complex
OS: Overall survival
PCNA: Proliferating cell nuclear antigen
pCR: Pathological complete remission
PD-1: Programmed cell death 1
PHH3: Phosphohistone-H3
p-JNK: Phosphorylated-mitogen-activated protein kinase 8
PLK-1: Polo-like kinase 1
PP2A: Protein phosphatase 2A
PR: Progesterone receptor
PTD: Protein transduction domain
RR: Relative risk
RTKN: Rhotekin
S-CDK: S-phase cyclin-dependent kinase
SCLC: Small cell lung cancer
SIX1: Sine oculis homeobox homolog 1
TBZ: Thiabendazole
TCGA: The Cancer Genome Atlas
TNBC: Triple-negative breast cancer
TOP2A: Topoisomerase IIA
TSA: Trichostatin A
